# A New DEM Generalization Method Based on Watershed and Tree Structure

**DOI:** 10.1371/journal.pone.0159798

**Published:** 2016-08-12

**Authors:** Yonggang Chen, Tianwu Ma, Xiaoyin Chen, Zhende Chen, Chunju Yang, Chenzhi Lin, Ligang Shan

**Affiliations:** 1 School of Environmental and Resource Sciences, Zhejiang Agriculture and Forestry University, Lin’an, 311300, Zhejiang, China; 2 Zhejiang Provincial Key Laboratory of Carbon Cycling in Forest Ecosystems and Carbon Sequestration, Lin’an, 311300, Zhejiang, China; 3 School of Information Engineering, Zhejiang Agriculture and Forestry University Lin’an, 311300, Zhejiang, China; University of California Los Angeles, UNITED STATES

## Abstract

The DEM generalization is the basis of multi-dimensional observation, the basis of expressing and analyzing the terrain. DEM is also the core of building the Multi-Scale Geographic Database. Thus, many researchers have studied both the theory and the method of DEM generalization. This paper proposed a new method of generalizing terrain, which extracts feature points based on the tree model construction which considering the nested relationship of watershed characteristics. The paper used the 5 m resolution DEM of the Jiuyuan gully watersheds in the Loess Plateau as the original data and extracted the feature points in every single watershed to reconstruct the DEM. The paper has achieved generalization from 1:10000 DEM to 1:50000 DEM by computing the best threshold. The best threshold is 0.06. In the last part of the paper, the height accuracy of the generalized DEM is analyzed by comparing it with some other classic methods, such as aggregation, resample, and VIP based on the original 1:50000 DEM. The outcome shows that the method performed well. The method can choose the best threshold according to the target generalization scale to decide the density of the feature points in the watershed. Meanwhile, this method can reserve the skeleton of the terrain, which can meet the needs of different levels of generalization. Additionally, through overlapped contour contrast, elevation statistical parameters and slope and aspect analysis, we found out that the W8D algorithm performed well and effectively in terrain representation.

## 1. Introduction

How to properly express and analyze terrain has always been the core of geography and is also the hotspot of cartography and geomorphology research. Because of the extreme complexity of the terrain surface, we cannot observe every detail of the Earth. Thus, the description of the terrain surface drawn by geospatial information is always approximate. This approximation can be seen as a scale of our observations of the terrain surface [1]. Thus, scale is one of the most important features of all the geographical information. Therefore, scale, as a basic concept, has interested many scholars.

The digital elevation model, DEM for short, is the digital simulation of terrain surfaces using limited terrain elevation data [2,3]. The specialized subject of geomorphometry has grown significantly in recent years as more and better DEMs have become available [4]. Additionally, different scales of the terrain elevation data are needed in different application fields and different research topics. When a rough scale is needed, it is necessary to reduce the redundant data by generalizing the original accurate DEM. Therefore, to satisfy the need, relevant institutions have built multi-scaled, multi-resolution DEM by manually generalizing the terrain surface. Although manual generalization can provide a more accurate DEM, it costs a large amount of human and material resources and takes too much time [5]. Thus, how to automatically generalize DEM has become an urgent problem that needs to be addressed.

In this article, a new method based on a watershed and tree structure is proposed to collect terrain feature points from grid-based DEMs. We define this method as a method that generalizes the terrain through an eight-direction radial line of each pixel on the border of the watersheds, and we call it the W8D algorithm.

The objectives of this study are (i) to assess the feasibility of W8D in selecting critical points and (ii) to compare the performance of W8D with the performance of widely used generalization methods, namely, aggregate, resample and VIP.

The paper is organized as follows. Section 2 reviews the terrain generalization methods. Section 3 introduces the study area and experimental data. Section 4 introduces a new method for terrain generalization. Section 5 gives the experiment results and evaluates the accuracy of the proposed method via experiments and comparison with other methods. A brief conclusion is given in Section 6.

## 2. Related Works

There are various methods of generalization. The methods of generalization based on DEM can be grouped into five categories, namely, regular grid, feature-point methods, point-additive, point-subtractive, and compound method.

The regular grid method is the simplest method for generalization; through resampling, a DEM model with low generalization accuracy is generated. The less points that are resampled, the more points are deleted, the coarser the generated DEM will be.

The feature point method uses a set of critical feature points, including peaks, saddles, ridges, valleys, and concave points. These feature points in every DEM can be recognized and categorized through certain algorithms [6]. In DEM, feature points are recognized through a 3*3 window. Using the feature points that are recognized, the terrain factors are analyzed; thus, the terrain properties are inferred. After TIN is constructed by extracting feature points, DEM can be reconstructed based on TIN to achieve terrain generalization [7]. For example, Chen and Guevara proposed the VIP algorithm, which measures the ‘significance’ of each elevation point by the difference between the actual elevation and the estimated elevation by the surroundings at the central point of the window [8]. Although the VIP method performed well, it depends heavily on the algorithms that use many indefinite specifications of tolerance. Furthermore, the method performs better in local processing, where it does not consider the global process [9].

The point-additive method can be further grouped into two categories: hierarchical subdivision [10] and refinement methods [10–12]. For example, in the maximum Z-tolerance algorithm, an original TIN, which is composed of 2 triangles, is constructed first. Then, the difference between every point in the elevation raster and the estimated elevation from the TIN is computed. The algorithm determines the point with the largest difference. If the difference is greater than a specified z-tolerance, the algorithm flags the point for addition to the TIN. After every triangle in the current TIN is checked, a new triangulation is recomputed with the selected additional points. This process continues until all points in the raster are within the specified maximum z-tolerance. The point-additive method can capture the overall morphological features very well, but badly misses the shape and topological relationships of drainage features, particularly in the flatter areas [13].

The point-subtractive method, which can also be called the drop heuristic method, is the reverse of the point-additive method in which the points in the triangles are gradually removed until it reaches the threshold of simplification [14]. For each iteration, the error at each grid point is computed, and the point that has the lowest error is removed. Although DHM performs well, it is very time-consuming, and it is not commonly used.

The compound method combines some of the methods mentioned above [13,15,16]. For example, Dong and Tang gave each elevation point an information index to achieve the grading of all points. They added the terrain structure lines base on the grading of the points. Then, a constrained TIN was formed. However, this method has the following defects: firstly, the terrain structure lines and feature points are in some extent coincident; secondly, the terrain structure lines that are extracted from the original DEM are not thoroughly generalized, which causes discordance between the generalization scale of terrain structure lines and the generalization scale of DEM; thirdly, the amount of points that will be obtained after generalization is difficult to control; thus, this method is not practical for purposes that are concerned about the amount of data [13,17]. Another example is a method that was proposed by Zhou and Chen. In this method, they first adopted the maximum z-tolerance algorithm to extract the points that are not on the stream network; then, they used the D8 algorithm to extract feature points and the stream network. Through the DP algorithm, they simplified the stream points and removed the coincident points. Finally, a constrained TIN of the stream network was formed. Although in this method, the coincident points are removed and the valley line is simplified, the process of extracting points is relatively more complex and involves double thresholds, which require more human-machine interaction and affect the actual implementation of the method. Furthermore, this method does not extract points on ridge lines when ridge lines are not constrained, which means the method needs further improvement to avoid the distortion in some small areas.

Although each method has its own redeeming features, most are based on the structural feature of DEM or TIN while, to some extent, neglecting the fact that the watershed is a geographic entity that exist objectively and that it is multi-scaled. It would be of great significance if a study could be conducted on the basis that a watershed is a geographic entity that exists objectively. Additionally, the widely used generalization methods we have are mainly based on the DEM grid and its neighbourhood. Additionally, these methods cannot indicate effectively the important level and the hierarchical quality of the terrain feature points. Thus, they are unable to realize a self-adaptive generalization.

The tree structure is a widely used data model. For example, the method of automated reasoning with contour maps proposed by Cronin adopted the contour containment tree model [18]. Roubal and Poiker considered the utilization and ascertainment of the relations between contours as the key to terrain generalization [19]. They proposed that a contour containment tree model, or contour tree, is needed during the generalization of contours. Hind T and Jean P conducted a research on the method of acquiring new contours by extracting the skeleton from the area of the contour interval, and they later used the method in the generation of DEM [20]. The construction of a tree model is helpful to grasping the features of the topography. Thus, this paper performed a terrain generalization study by adopting a tree structure representing the multi-scaled watershed.

## 3. Study Area and Experimental Data

**Fig 1:**
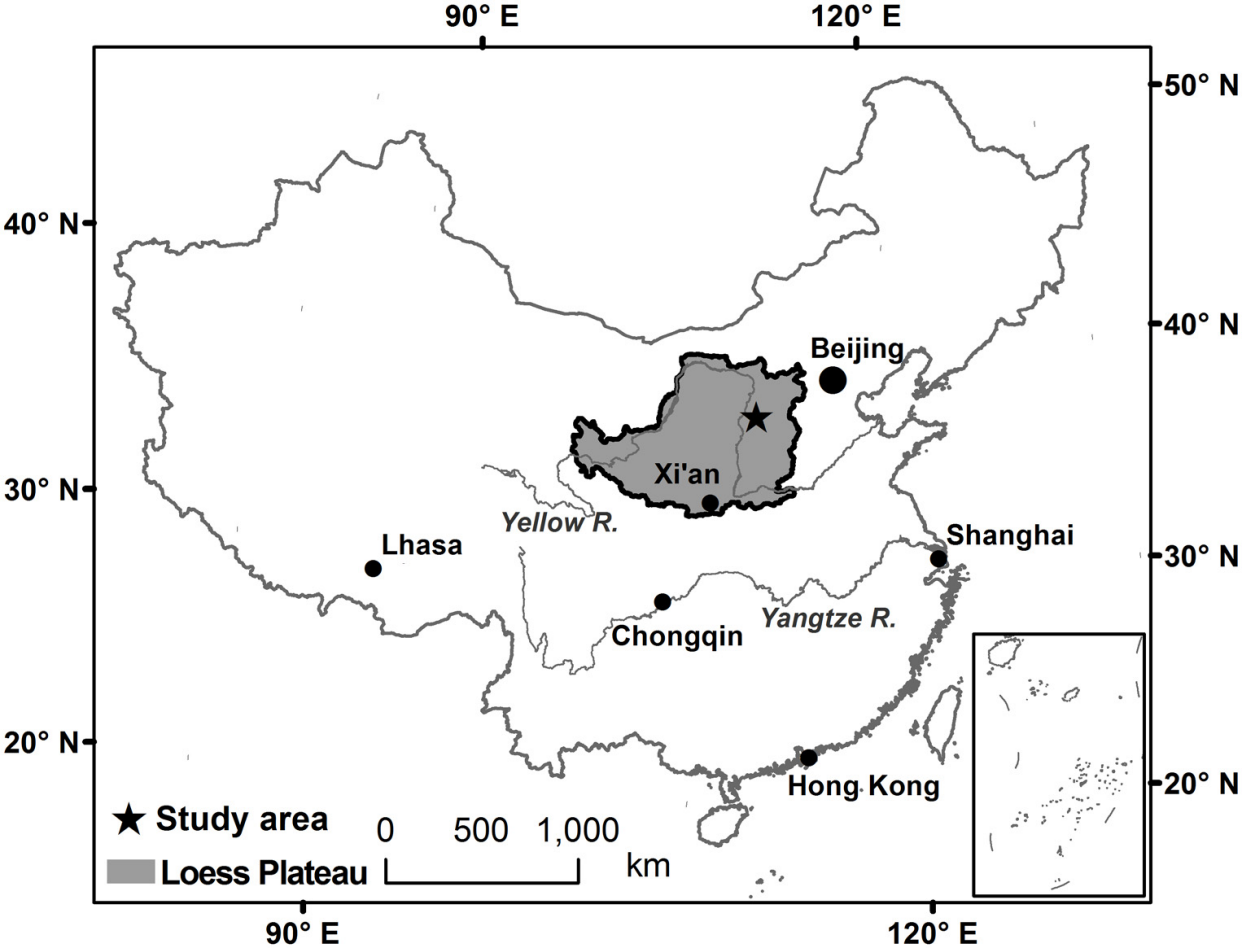
Location of the study area.

The study area is located in Suide County, Shanxi Province, China. The geographical position for the area is east longitude 110°15´00″—110°22´30″, north latitude 37°32´30″—37°37´30″, and the altitude is 814–1188 m. The area covers 70 km^2^, with the altitude ranging from 832 to 1200 m. The loess crisscross gully and hillock gully are fully developed in this area, knows as the loess hilly and gully region, with an average slope of 29.3°. The location of the study area in China is shown in Fig 1. DEM of study area is performed with a grid size of 5 m, which is comparatively precise and can be used to extract and analyze the detailed features of small watersheds.

## 4. Theories and Methods

**Fig 2:**
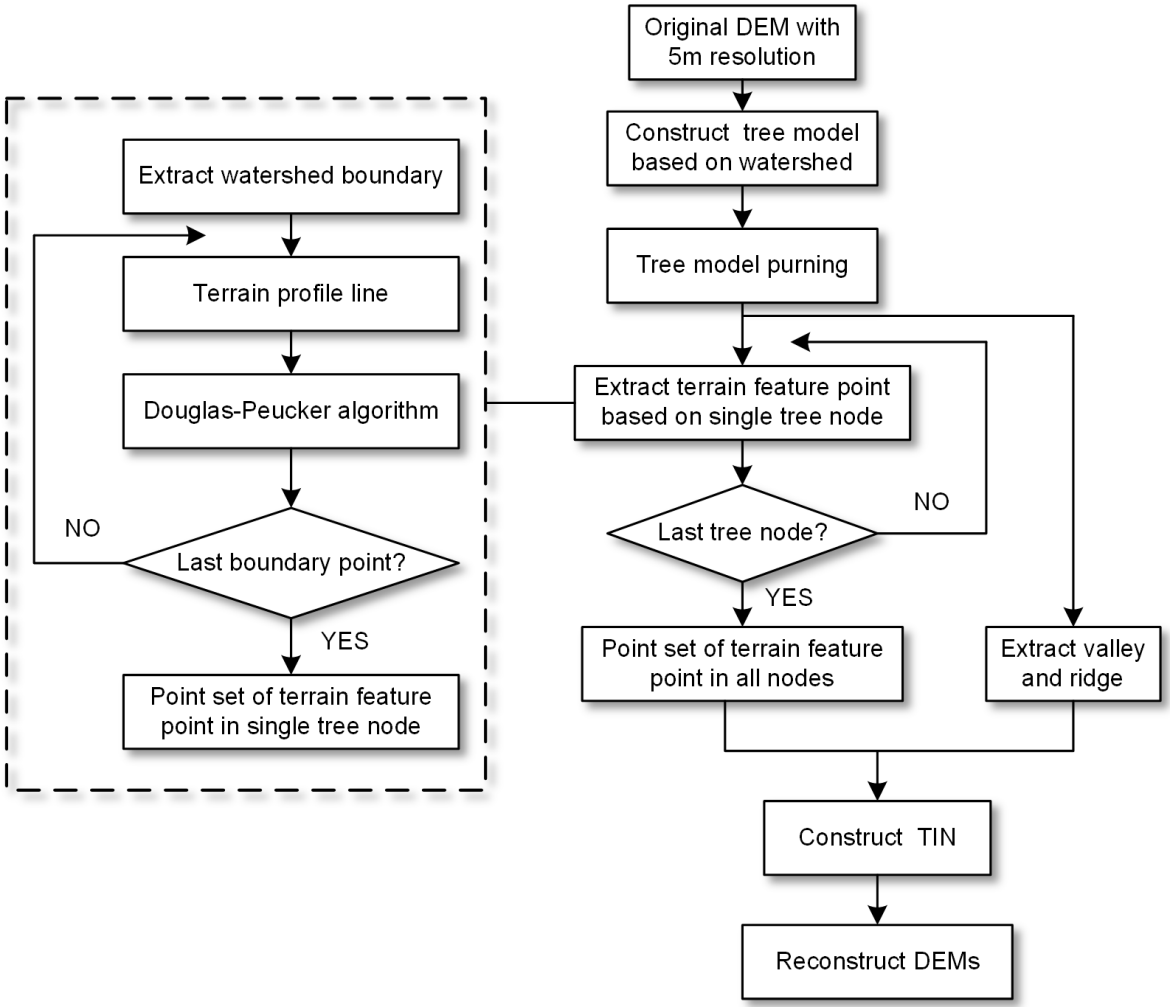
Working flow of DEM terrain generalization.

The W8D algorithm aims to generalize high-resolution DEM. The major steps are: construct tree model, pruning, generate eight-direction radial line, extract and generalize feature points using the Douglas-Peucker algorithm, construct a triangular irregular network and generate the generalized DEM through interpolation. Fig 2 is the procedure of terrain generalization based on DEMs.

### 4.1. Tree Model Construction

**Fig 3:**
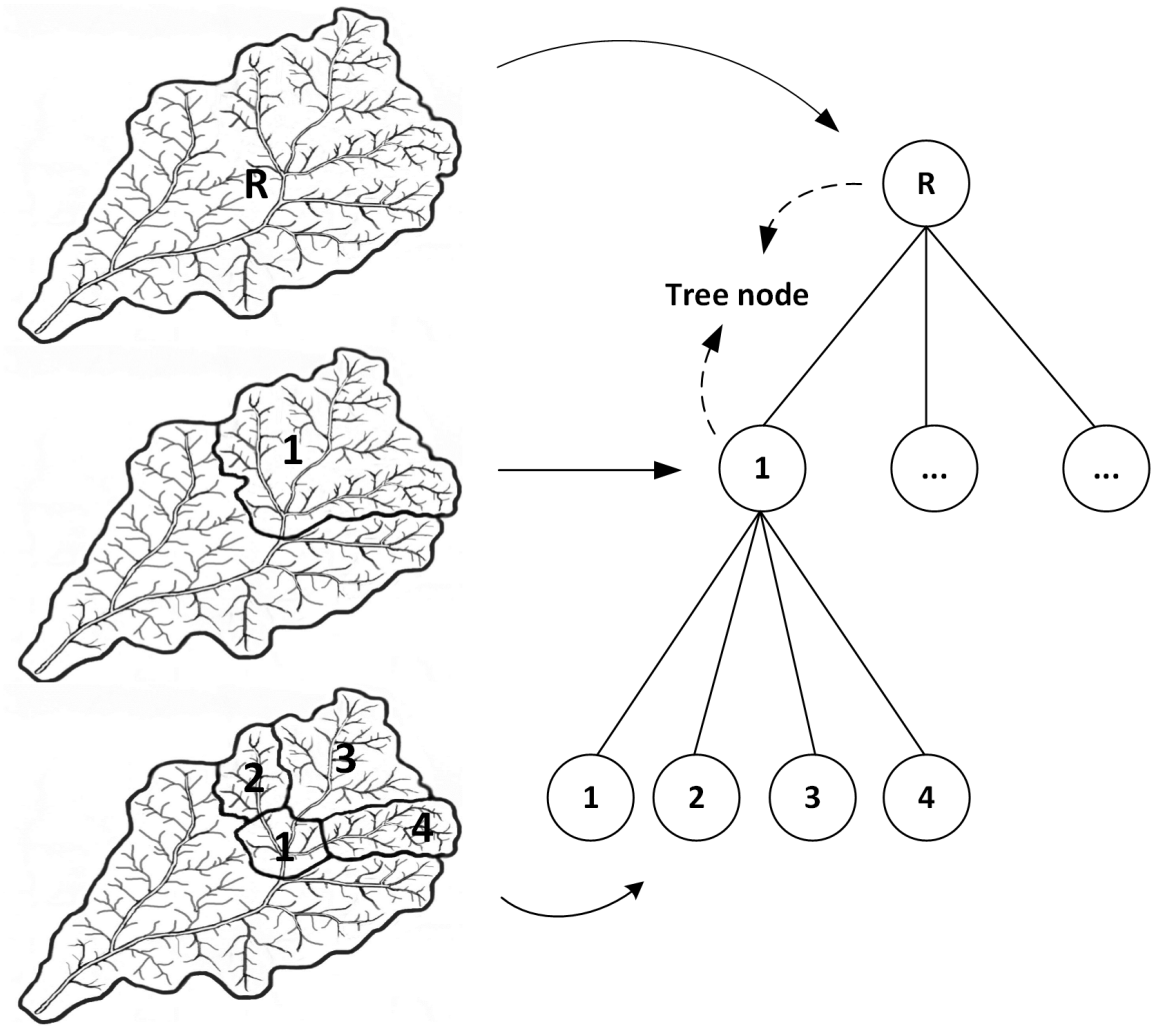
Schematic diagram of the tree structure creation.

A tree model structure represents a hierarchy in a graphical form composed of nodes and lines, and can be used in various scientific fields including geography(Chen et al. 2016). The nodes is the node of the tree, and the lines that link nodes together are branches. The tree model can better describe hierarchical relations, subordinate relations and coordinate relations. Delineation of watersheds can take place at different spatial scales [21,22]. A large watershed might cover the whole river system. Within the watershed, a small watershed might entail a smaller watershed. Each watershed is a tributary of the river system. It is the nesting feature of a watershed that makes it possible to simulate the watershed structure through tree model. When looking at the tree as a whole, each node on the tree contains the relevant information about the watershed. Different hierarchies represent different observation scales. We can consider the relationships between a parent watershed and a child watershed as the relationships between a large watershed and a small watershed. Small watersheds together form a large watershed, with smaller watersheds entailed in them. Thus, the total of the nodes can be seen as the whole study area. The total of different levels of nodes represents different scales. The watershed information in each node is flexible and controllable by the researchers. The branches link the different levels of nodes together. The tree structure model, as shown in Fig 3 in this paper, is founded on spatial inclusion relations and is formed by different scales of watershed.

Different levels of watersheds are extracted using the classic D8 algorithm based on different scales [23]. The combination of these extracted watersheds forms a watershed tree model. These extracted watersheds from different levels are the layouts of the watershed terrain that is contained in the tree nodes. How many watersheds are distributed to each tree node is decided by the watershed areas. Thus, the hierarchical relations, the subordinate relations and the coordinate relations are presented. The maximum threshold used to define a watershed is important. The U.S. Environmental Protection Agency has given the definition that the maximum area of a small watershed is 77.7 square kilometres. Meanwhile, according to the Institute of Mountain Hazards and Environment, CAS, when the area is smaller than 0.2 square kilometres, it is difficult to form a catchment area [24]. Thus, in the paper, the maximum area of a watershed represented by the root node cannot be more than 77.7 square kilometres, and the smallest area of a watershed represented by the leaf node cannot be less than 0.2 square kilometres. In other words, the initial threshold and the termination threshold of a watershed are 0.2 square kilometres and 77.7 square kilometres, respectively. If a small increment is set, through circulative iteration, we can repeatedly segment the watersheds. As long as the increment is small enough, we can extract every small watershed from each flow accumulation value. When the watershed in every level is extracted, a suitable watershed will be chosen as the root node of the tree model. During the construction of tree model, some fake nodes might be formed. A fake node is a watershed that appears in the neighbouring levels of the tree model. A fake node often covers the same amount of area and overlaps with the neighbouring node. To delete the fake node, we adopted the methods of manual selection and exhaustion. An intact tree model is constructed when all of the fake nodes are deleted.

### 4.2. Global terrain generalization based on tree model pruning

When the construction of the tree model is complete, pruning is performed, that is, rejection of the small watersheds. An intact tree model can represents the whole watershed system on every scale. Global pruning is used to reject the watersheds that are not in the range of the threshold while guaranteeing the intactness of the tree model.

**Fig 4:**
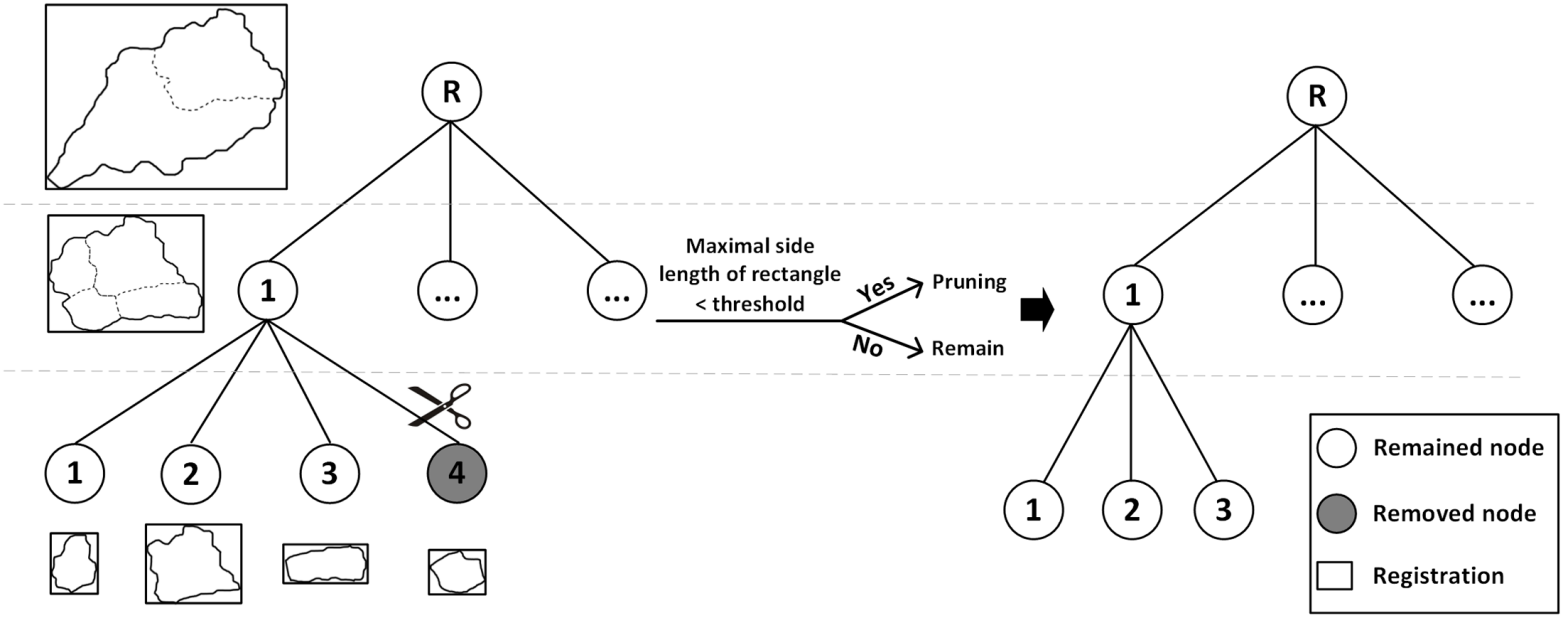
Example of a watershed tree for pruning.

The pruning algorithm used in the paper is adopted from decision tree. The major function of decision tree is to explain structured information of data and is thus similar to the tree model in our research. The pruning of decision tree can be grouped into pre-pruning and post-pruning. To build a fully grown tree model so that the relationships between tree model and multi-scaled watershed can be presented, the author adopted the method of post-pruning. However, the selection of the best splits is based on increasingly smaller samples as the tree grows. The spilt choices at the lower levels of the tree often become statistically unreliable, and the tree is said to overfit [25]. When the tree model is fully developed, certain criteria are adopted to reject some subtrees of the tree model. Fig 4 shows an example a watershed tree for pruning. Due to the irregular shape of watersheds, the area is difficult to measure. Thus, the paper used the longest edge of the boundary rectangle to measure the watersheds. In this way, the calculation is more convenient. A boundary rectangle is used to fit every watershed shape that is represented by every tree node. Then, a traversal of all of the tree nodes is conducted. When the longest edge of rectangle is longer than the selected pruning threshold, the node is reserved; otherwise, the node and its subtree are deleted. The watersheds that are represented by them are also rejected. In Fig 4, assuming the longest edge of the rectangle of node 4 is shorter than the selected threshold, node 4 is deleted.

### 4.3. Global terrain generalization based on tree node

The previous part presented the method of global generalization, that is, pruning on the premise that the tree model is fully developed. Nodes, as a whole of the watershed, are involved in the global pruning. However, this section presents the method of generalization towards a single watershed. Speaking from the perspective of tree model, it is a generalization that is conducted towards a single node. This generalization involves all of the nodes and is different from the previous global generalization but is conducted on the premise that the global generalization has been performed. In conducting this generalization, our focus is laid on the extraction of the feature points. We extract the feature points of watersheds from every level of the tree model and then overlay the layer that we obtain from extracting every level of the watersheds from the tree model. Finally, we obtain all the feature points from every scale and can use them to reconstruct the DEM in the target scale.

#### 4.3.1 Terrain generalization based on a single tree node

**Fig 5:**
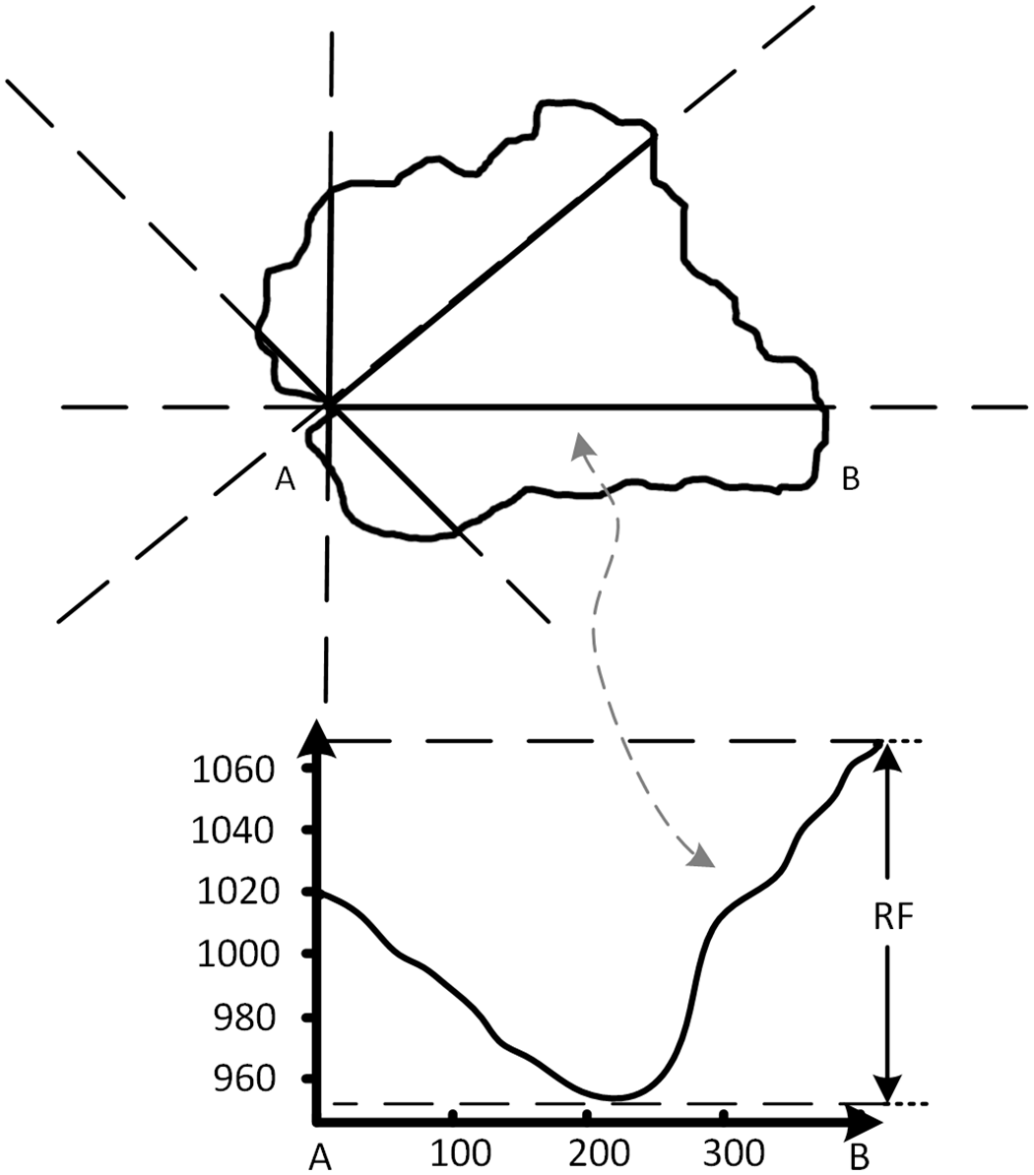
Schematic diagram of generating terrain profile lines in one direction.

4.3.1.1 The principle of the 8 direction terrain profile simplification: As shown in Fig 5, a point that is located on the watershed boundary represented by a single node from the tree model is chosen, and a radial line is drawn from this point in eight directions: east, west, south, north, southeast, southwest, northwest, and northeast. If there is no watershed area in the direction of a radial line, then the radial line is rejected. If the radial line runs through the watershed area, it is reserved. The radial line stops extending when it reaches the boundary on the other side of the watershed. In this way, a single point on the boundary of a watershed can at most have eight radial lines. When we obtain the elevation value from the radial lines that passed through the watershed and convert the feature to 3D, the terrain section line of the eight directions from one single point is presented. The terrain section line can present the changes of the terrain surface very well from one point to the other. In these terrain section lines, the vertical axis is the elevation value, and the RF in the profile graph is the largest elevation difference value in this line. The horizontal axis is the central coordinate of the grid points on the radial line. Then, we use the Douglas-Peucker algorithm to perform linear simplification to further accomplish the extraction of the feature points. The Douglas-Peucker algorithm has become a major method for automatic generalization towards linear features in computer cartography and GIS studies [26]. The Douglas-Peucker algorithm can extract feature points in curves and then form a new generalized line according to a target scale. The feature of this algorithm is that it can select the feature points that represent both the general shape and the local features of a curve through a relatively simple global recursive operation [27].

**Fig 6:**
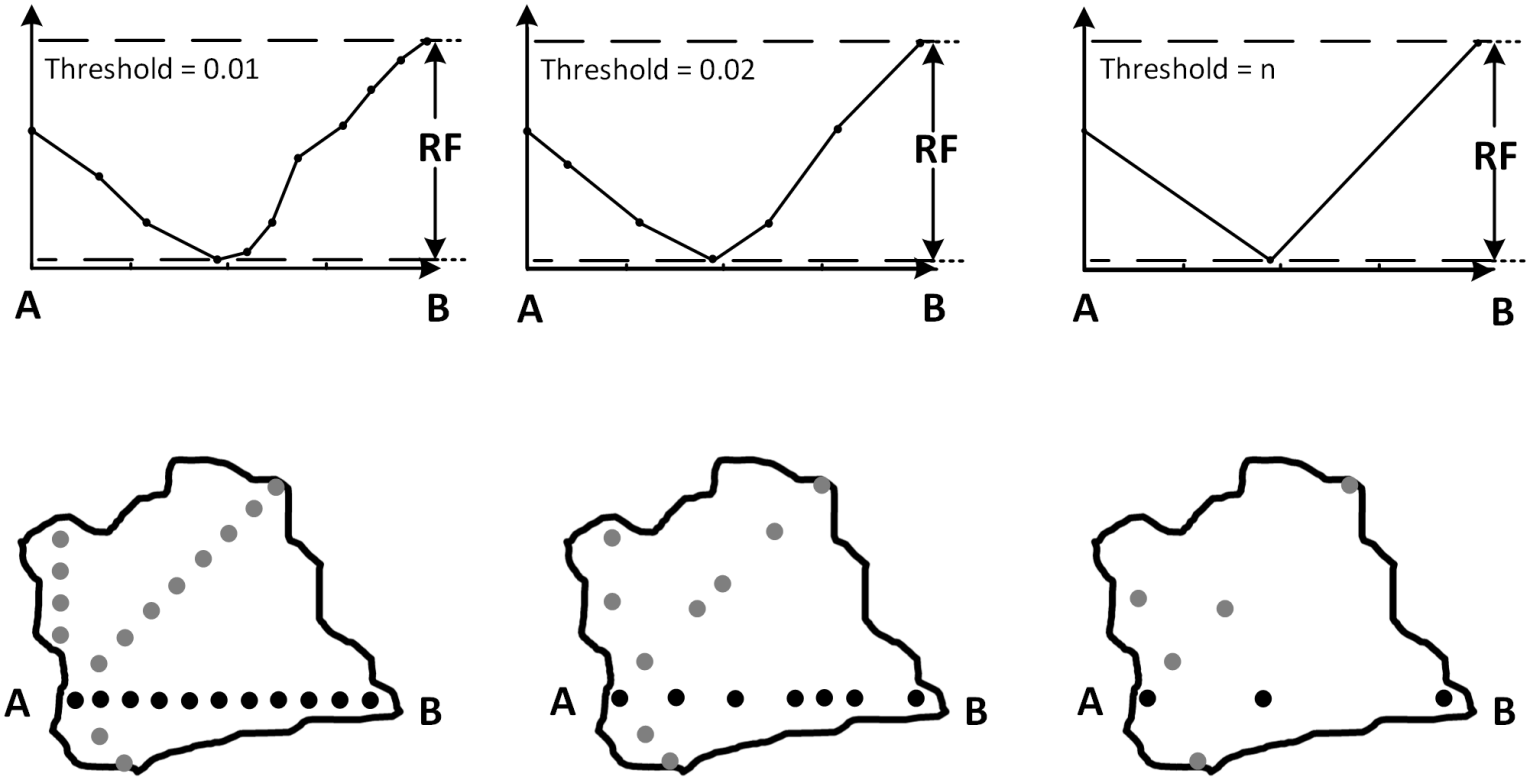
Schematic diagram of terrain profile line simplification.

4.3.1.2 Selection of the W8D algorithm threshold: Simplifying the threshold is the key of the W8D algorithm and directly influences the outcome of generalization. The W8D algorithm and the Douglas-Peucker algorithm are complementary. The threshold of the Douglas-Peucker algorithm is decided by both the threshold of the W8D algorithm and the height deviation of the to-be-simplified section line. The threshold of W8D represents the degree of terrain simplification. The value range is (0,1). The relationships of the threshold of the W8D algorithm, the threshold of the Douglas-Peucker algorithm and the height deviation of the section line can be stated as follows:

θ=T⋅RF

where θ is the threshold of the Douglas-Peucker algorithm, T is the threshold of the W8D algorithm, and RF is the height difference between the highest and the lowest point in the section line of a certain direction. Because the RF values of the eight directions in different border points are different and because the RF values of the same border point in different scales are different, the values of θ in the Douglas-Peucker algorithm are different. In this way, we can reserve the feature points according to every individual section line, and we can conduct the generalization in a self-adaptive and a self-explained way. The value of T is in the range of (0,1); researchers or users can select the most proper threshold according to the needed generalization scale. The closer T is to 1, the higher the generalization scale. With the change of threshold, the number of points in the section lines decrease, and the feature points are reserved (Fig 6).

**Fig 7:**
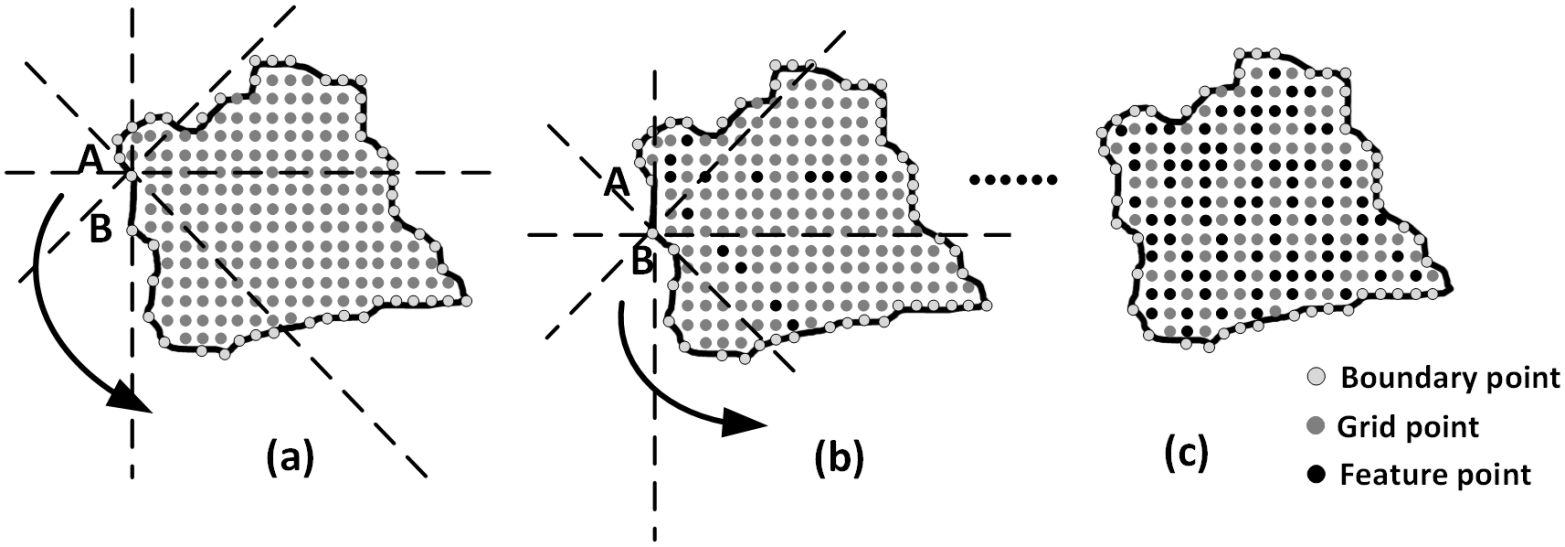
Boundary points traversal of the W8D algorithm.

4.3.1.3 Traversal of points on the watershed border: The W8D algorithm is used on every border point of the single watershed. When the traversal of every border point is complete, we obtain all of the feature points in the watershed. Then, the W8D algorithm is used on every node in the tree model, and the feature points in the whole watershed are presented. Meanwhile, some feature points in the same watershed might repeatedly appear and must be rejected. The following reason might introduce this problem: as shown in Fig 7, in the same tree node, the generalization of different border points from eight directions might present some of the same feature points.

#### 4.3.2. Global terrain generalization based on the tree structure

The previous sections presented the terrain generalization towards a single watershed. This section will complete the global generalization, that is, the traversal of every node of the tree structure. The pseudo code of this section follows (Box 1). treeNode is a node on the tree structure, edge is a branch on the tree structure, shpWatershed is the terrain skeleton inside the watershed, DEM is the original DEM data, and fPoint is the feature points that are extracted from watersheds.

**Box 1. The pseudo code of global terrain generalization based on the tree structure**

1:**for each** node in WatershedTree **do       //**node means watershed

2: boundary = BoundaryOfWatershed(node)   //boundary means boundary of watershed

3:  **for each** vertex in boundary **do     //**vertex means point of boundary

4:   fPoints = W8D(vertex)

5:  **end for**

6:**end for**

**Fig 8:**
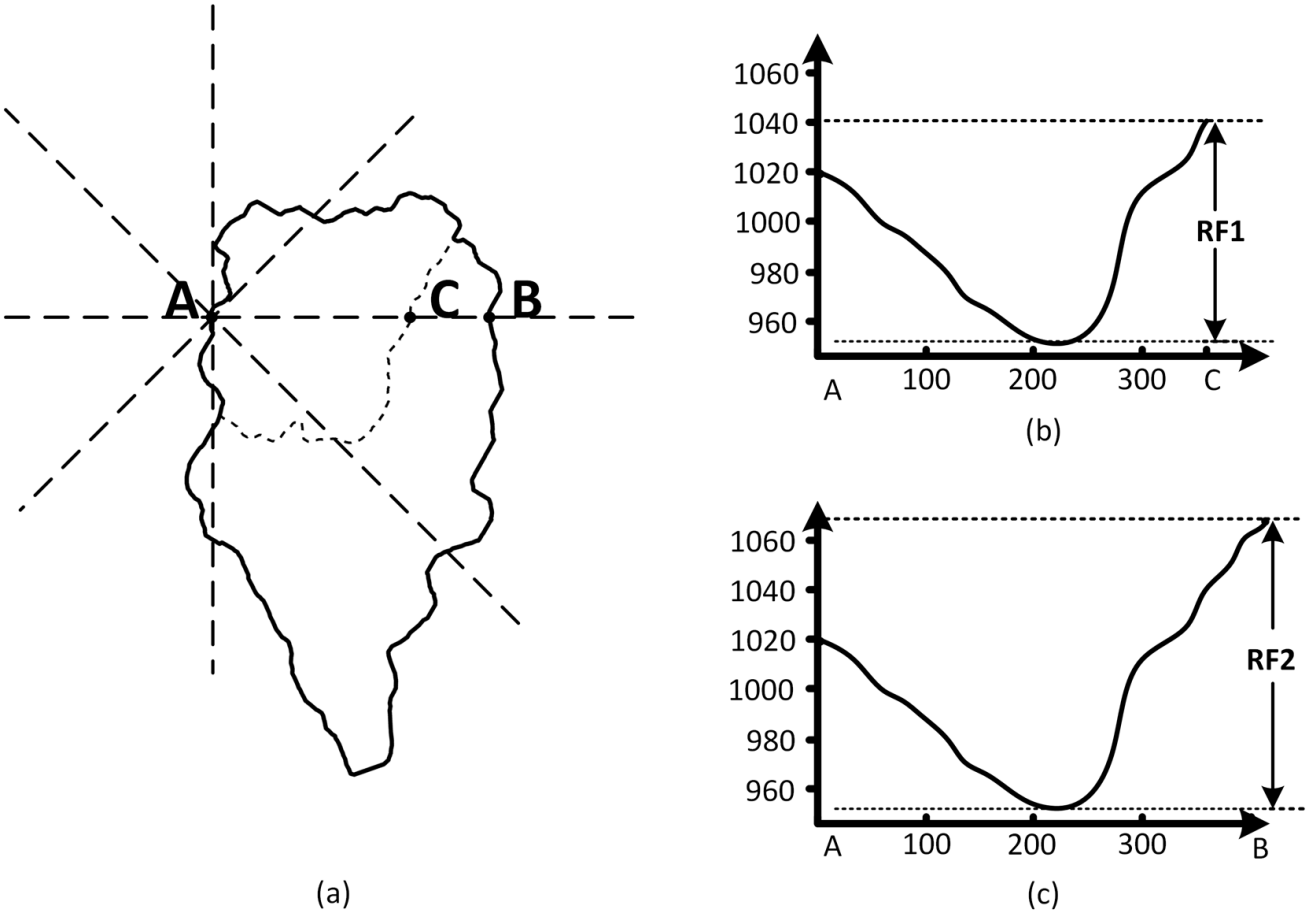
Boundary points traversal of the W8D algorithm.

In the tree structure, the nesting relations enabled the feature points of the same small watersheds to be extracted from different tree levels. Thus, there are two reasons that some feature points are repeatedly extracted. As shown in Fig 8, the repeatedly extracted feature points may come from both watersheds represented by root nodes and watersheds represented by a child node. The second reason is the different boundary point for using the W8D algorithm in the same watershed can generate the repetition. These repeated feature points can be deleted by overlapping the watersheds. By placing all of the feature points on the same layer, we can obtain all of the feature points in the whole tree, or, in other words, in the whole watershed. Moreover, as is shown in Fig 8(b) and 8(c), a nesting watershed in the same direction may create the different RF, which means the selection of the feature points is different under different levels of tree models, and the higher level can contain more information.

### 4.4. DEM reconstruction

After the W8D algorithm is used on every tree node, we obtain the feature points in every watershed of every scale. Because the skeleton feature of the watersheds is also represented by the tree nodes, we can use the skeleton of the watersheds to construct a constrained TIN and then reconstruct DEM. There are several methods of interpolation to construct the terrain surface, but the most common method to generate DEM is still first constrained TIN and then using the method of interpolation to convert TIN to DEM. The breakline is abstained from the D8 algorithm to avoid the problem of triangle-crossing skeleton lines. To improve the quality of the DEM that is converted from the TIN, we adopt the natural neighbour technique, which can achieve a smoother interpolation. Finally, we obtain the DEM under different thresholds.

## 5. Result and Analysis

### 5.1. Experiment result

**Fig 9:**
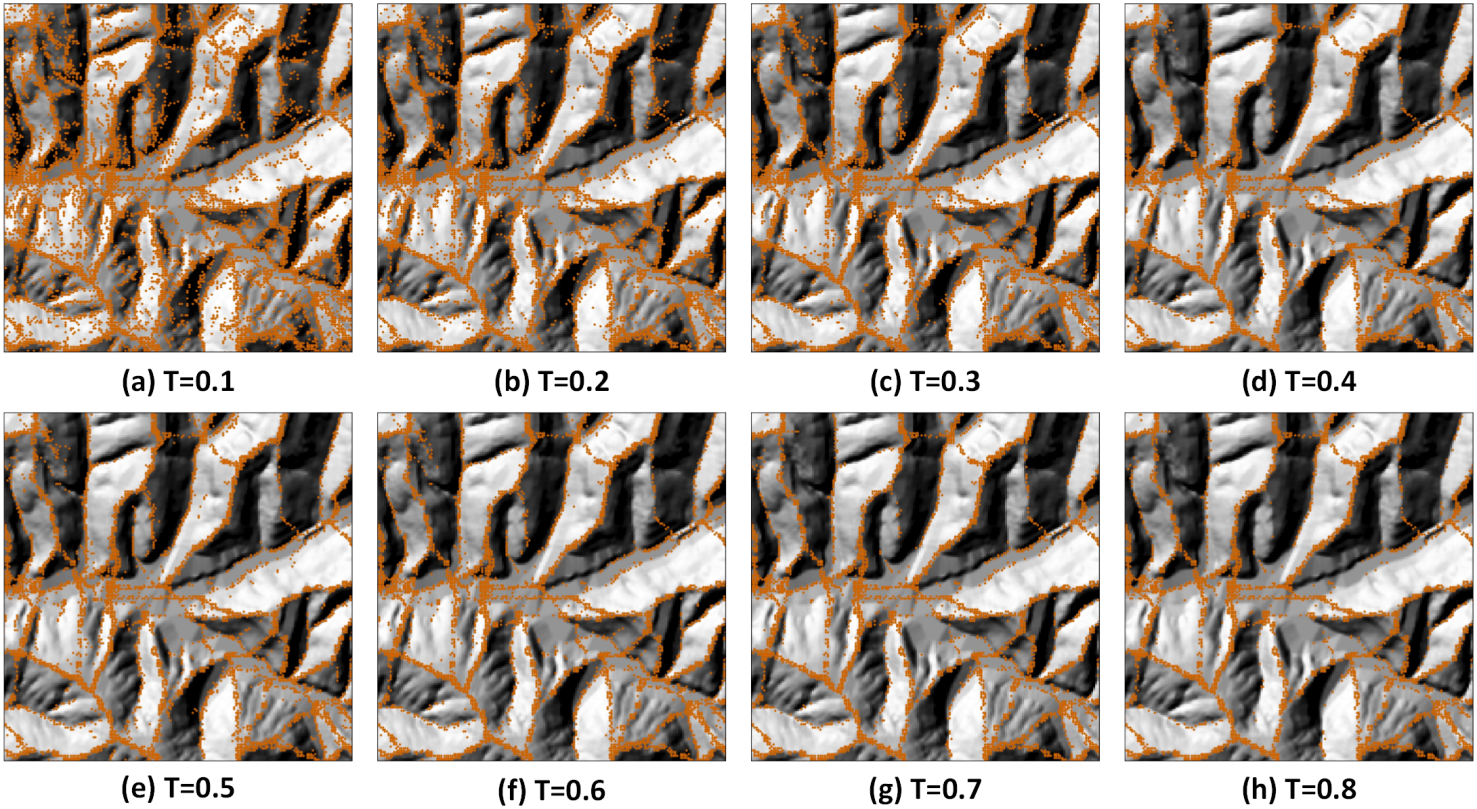
Spatial distribution of the terrain feature points under different thresholds. The basic DEM image is original didn’t generalization and the dots in orange are the terrain feature points.

In this section, we use the 5 m resolution DEM of Jiuyuan gully to conduct the generalization of the tree structure. By setting different thresholds in the W8D algorithm, we can extract the feature points from nodes of different levels. Fig 9 shows the feature points that are extracted under different thresholds.

**Fig 10:**
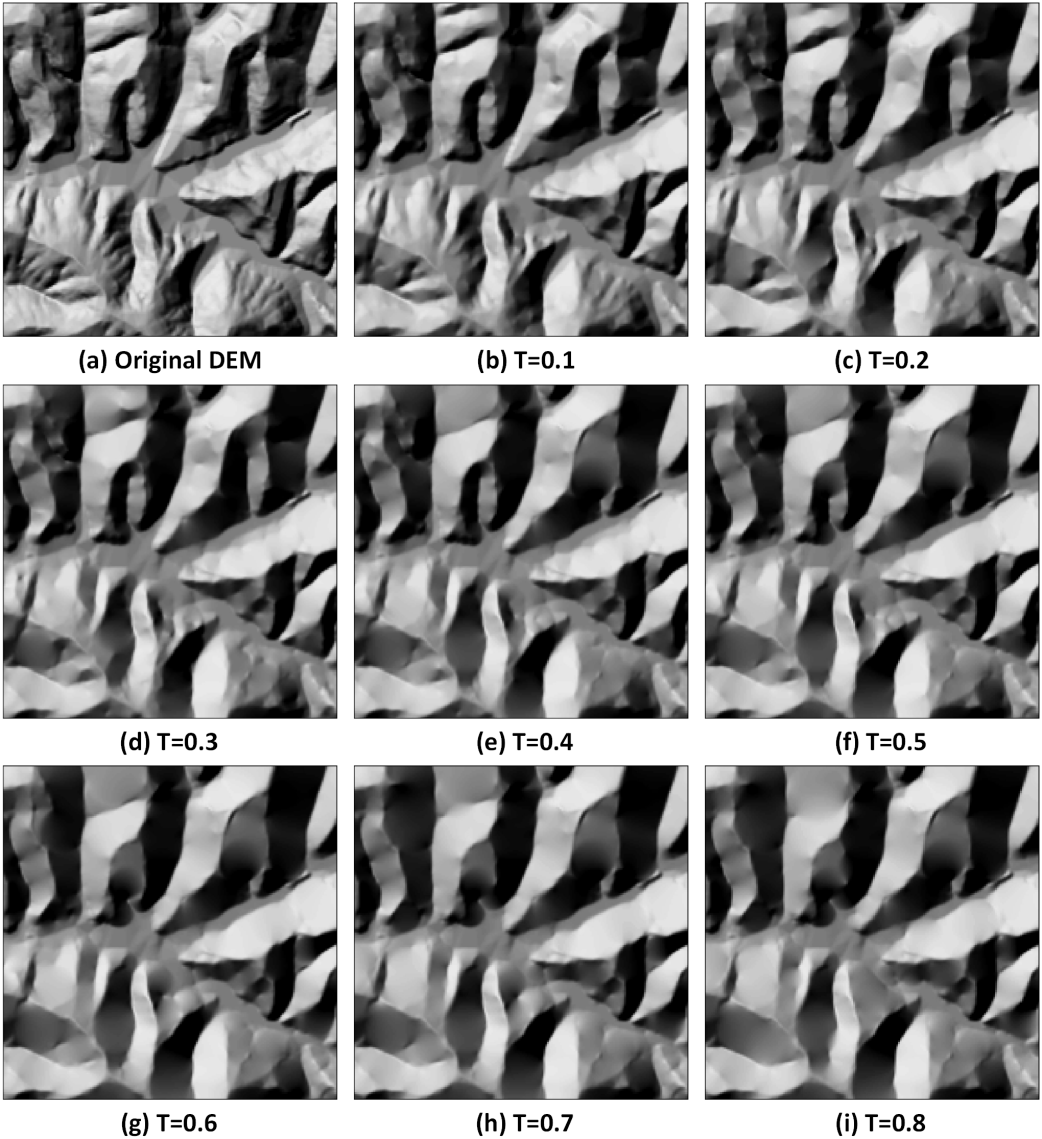
Results of the generalized DEMs with different thresholds.

As we can see from Fig 9, when T, the threshold, is smaller, the detailed feature points that are between the valley lines and ridge lines are reserved; thus, the detailed features of the area are presented. When T is larger, the small gully, ridge, and the detailed small points are gradually simplified, whereas the larger and more obvious feature points that represent the whole structure of the area are reserved. Then, we can reconstruct the generalized terrain surface through interpolation using these reserved feature points. The TIN and the DEM that are generated by adopting natural neighbour technique are shown in Fig 10.

**Fig 11:**
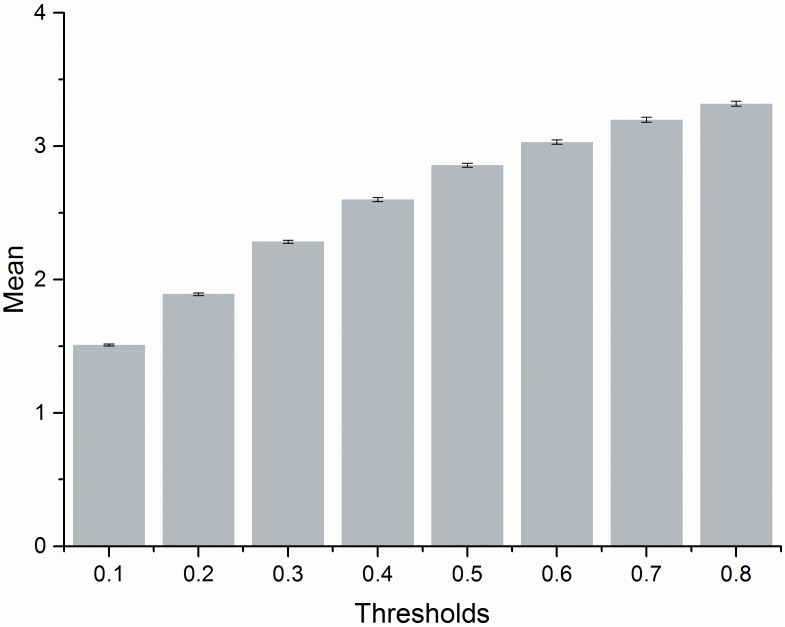
A comparison of the mean difference for the 5 m resolution DEM under different thresholds. The y-axis according to mean difference and error bars according to SE Mean in Table 1.

We calculated the differences between the reconstructed DEMs that are generated under different thresholds and the original 5 m resolution DEM cell-by-cell and then determined the absolute value of the differences. We recorded the mean absolute value of the height differences of the cells before and after the generalization and the standard deviation (Table 1 and Fig 11). The standard error of the mean (SE Mean) estimates the variability between sample means. Use the standard error of the mean to determine how precisely the mean of the sample estimates the population mean. The SE Mean can be as computed as follows:

$$SE\;Mean=\frac{SD}{\sqrt{N}}$$

Where SD is the standard deviation of elevation differences and N is the number of cell on DEM.

**Table 1:** The accuracy assessment of the generated DEM against the 5 m resolution original DEM.

T	0.1	0.2	0.3	0.4	0.5	0.6	0.7	0.8
Mean difference	1.510	1.890	2.284	2.600	2.856	3.030	3.198	3.318
SE Mean	0.007	0.010	0.012	0.015	0.016	0.017	0.018	0.019
Sample number	35417	35417	35417	35417	35417	35417	35417	35417

Table 1 and Fig 11 show that with the increase of the threshold used in the W8D algorithm, the generalization of the area grows more comprehensive. The difference between the 5 m resolution DEM and the generalized DEM becomes more obvious, whereas the standard deviation of the mean absolute value of the height differences stays low, with small variation. This result indicated that the outcome of W8D algorithm is very stable, whereas the terrain features are very well-reserved in the DEM dataset. Determining the value of threshold is the key step of generalization through the W8D algorithm. The threshold is determined by the target scale of generalization. Because it is affected by the complexity of the to-be-generalized terrain itself, the threshold chosen for generalizing different types of terrains will differ greatly. As we can see from Fig 11, there is not necessarily a quantitative relation between the chosen threshold and the scale of the terrain generalization. Thus, the threshold should be selected according to the terrain feature and the target generalization scale. The feature points should be extracted under different thresholds to construct the DEM. Through evaluation and analysis towards the generalized DEM, we can select the threshold that can best present the terrain features of the study area through the W8D algorithm.

The paper used 25 m resolution DEM dataset as the target scale to study and analyze the subject of how to determine the threshold value. We first produced the generalized DEM datasets generated under different thresholds, then computed the mean absolute value and its standard deviation of elevation differences cell-by-cell between the generalized DEM and the 25 m resolution DEM (Table 2 and Fig 11).

**Table 2:** The accuracy assessment of the generated DEM against the 25 m resolution original DEM.

T	0.02	0.04	0.06	0.08	0.10	0.12	0.14	0.16	0.18	0.20
Mean difference	4.864	4.859	4.856	4.860	4.870	4.868	4.893	4.905	4.925	4.954
SE Mean	0.017	0.017	0.017	0.017	0.017	0.017	0.017	0.018	0.018	0.018

**Fig 12:**
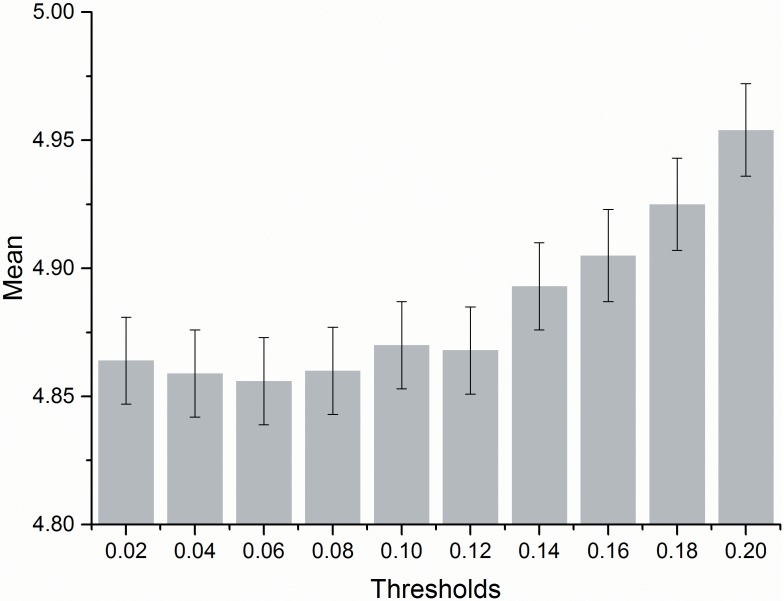
A comparison of the mean difference for the 25 m resolution DEM under different thresholds. The y-axis according to mean difference and error bars according to SE Mean in Table 2.

Fig 12 shows the regular change of the mean absolute value of elevation differences when the threshold increases, which is characterized by U curve. When the threshold increases from 0.02 to 0.06, the value decreases, and the mean difference stays below 5 m. When the threshold increased from 0.06 to 0.20, both the mean absolute value and its standard deviation increase as the difference rises, which indicates that with the increase of threshold, some small terrain feature points are neglected. According to both the 25 m resolution DEM accuracy and the terrain features of our study area, a difference of elevation below 5 m is within the allowed difference of the process of generating 25 m resolution DEM. Thus, in our analysis, a threshold of 0.06 is the best threshold for generalizing the 5 m resolution DEM to 25 m resolution DEM.

### 5.2. Comparison with other methods

#### 5.2.1. Classical generalization methods

There are several methods used to auto-generalize DEM; the most commonly used are the aggregation method, resample method and VIP method. This section used these three methods and the W8D method to generalize the same DEM; the target scale is 1:50000. In this way, the effectiveness and usability of the W8D algorithm are discussed.

The aggregation method creates a lower-resolution DEM from the original DEM. Each cell value in the target DEM is the mean value of an R×R window from the original higher-resolution DEM. This method is similar to the filtering technology in image processing. For example, in the 5 m resolution DEM, the mean value of 25 cells is the cell value of the 25 m resolution DEM, that is, the mean value of 5×5 cells is used as a single cell value in the 25 m resolution DEM. This is the principle of aggregation.

The resample method is a process that forms a lower resolution DEM by resampling points at equal intervals. The smaller the number of resampled points, the larger the number of points deleted during generalization, and the lower the resolution of the target DEM. Different methods of selection in the original DEM will lead to different resample outcomes. For example, when generalizing DEM from 5 m resolution to 25 m resolution through resample methods, we need to select a point in the original 1:10000 DEM. However, this point is the common point of 4 neighbouring cells. To collect the value, the value of any of these 4 cells can be resampled as the value in the 1:50000 DEM.

**Fig 13:**
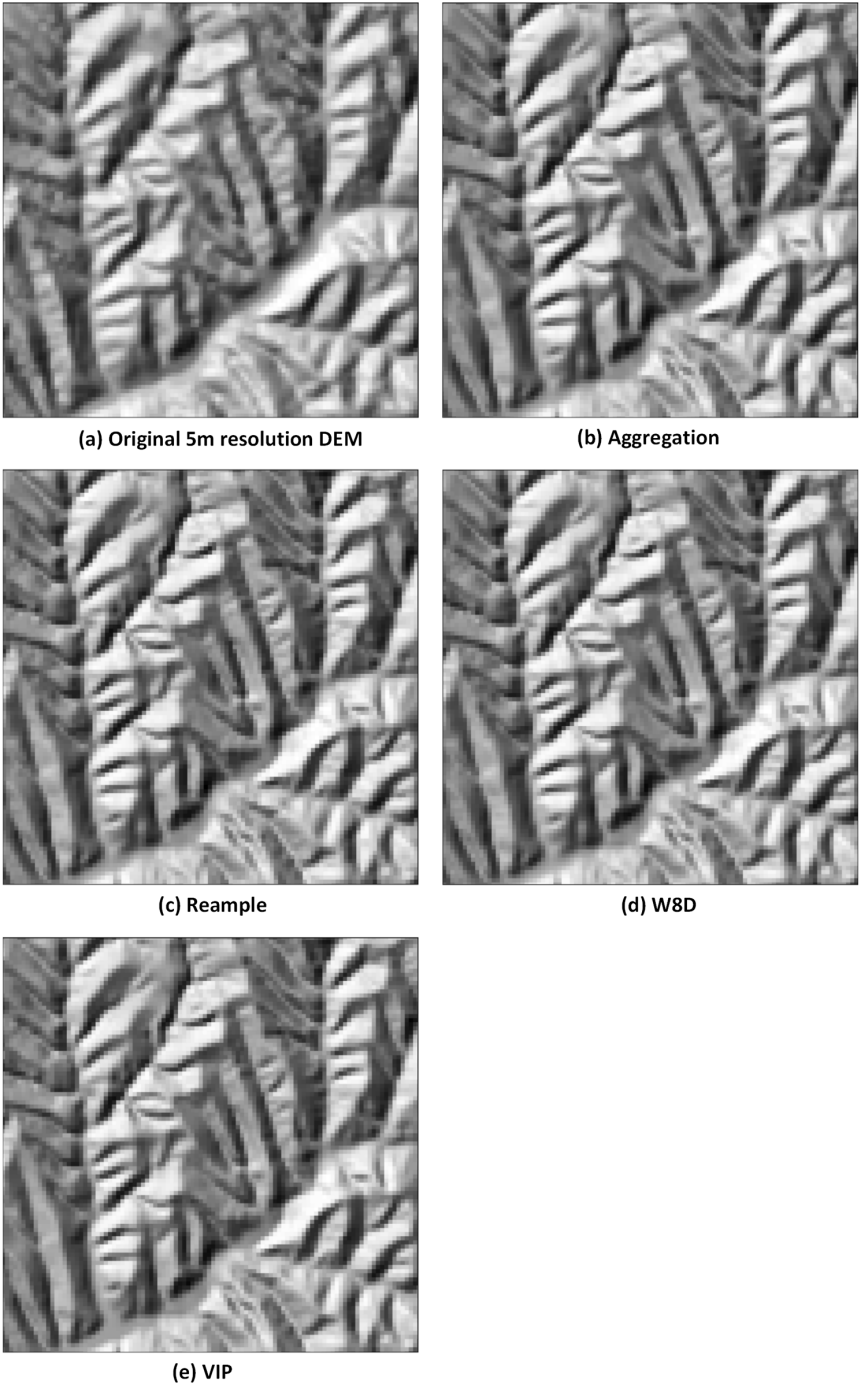
The comparison diagram of the different algorithm-generated DEMs enhanced the local details.

The VIP method reserves some important points to generalize the DEM. VIP selects points according to the roughness within the local grids. The importance of the grid determines whether the grid is reserved. The VIP method is usually conducted using a 3×3 moving window. By calculating the neighbouring eight cell value and the elevation differences, whether the central cell is important enough to be reserved is determined. The comparison diagram of different algorithm-generated DEMs enhances the local details (Fig 13).

#### 5.2.2. Comparative analysis

To assess the effectiveness and usability of the W8D algorithm, it is compared with other commonly used methods using different evaluation methods. These methods are: overlapped contour contrast, elevation statistical parameters, slope and aspect analysis.

(1) Overlapped contour contrast

**Fig 14:**
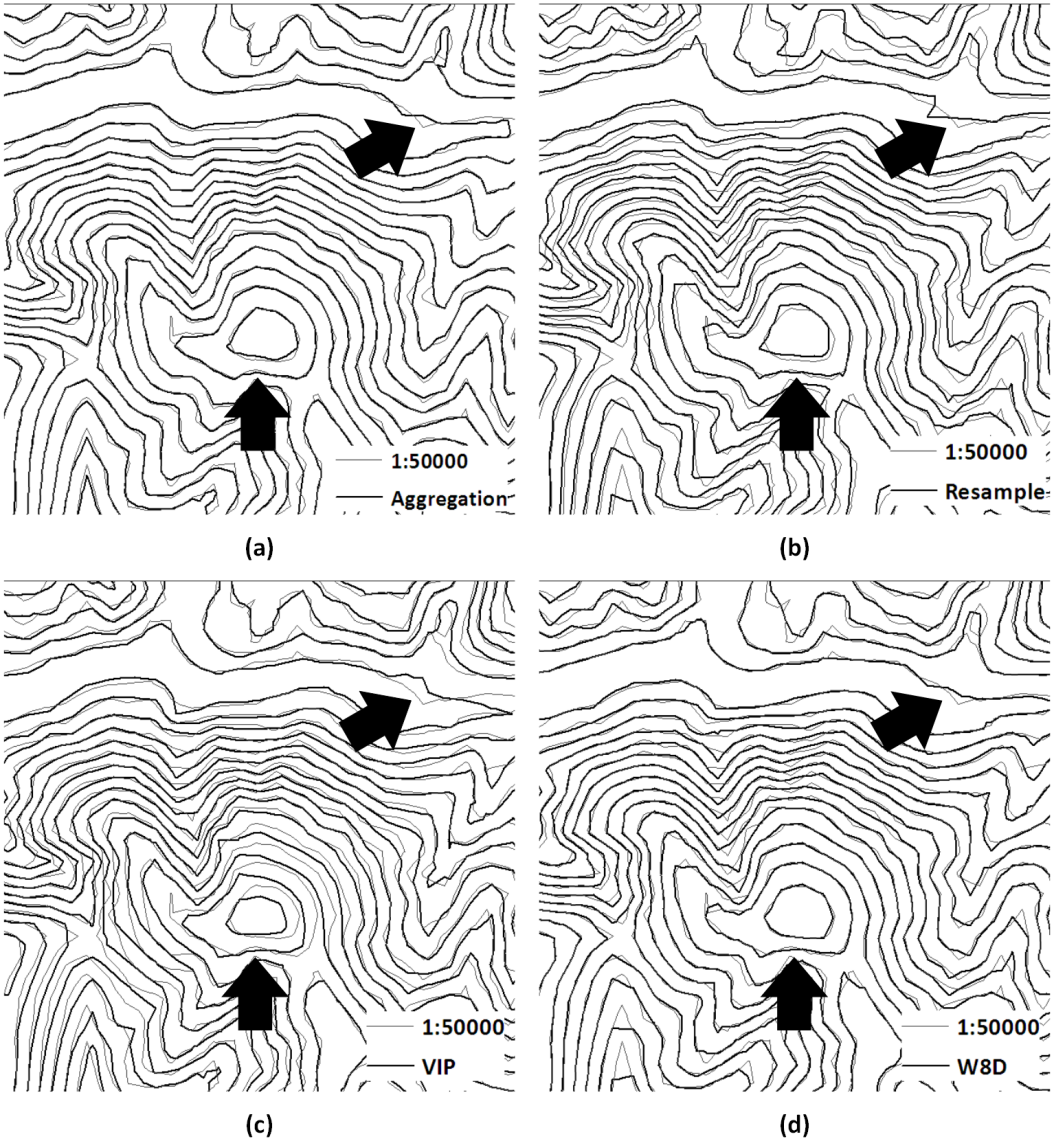
Overlapped contours extracted from the reconstructed DEMs. The thin lines are the contours that are converted from the actual 1:50000 DEM, whereas the thick lines are converted from the 1:50000 DEM and generalized from the 1:10000 DEM through different methods. And the black arrows pointing to the regions indicate the examples of effectiveness of overlaps using difference algorithms.

The displacement, the coinciding parts and the local conditions of the overlapped contour can indicate the effectiveness of the generalization method. In Fig 14, the contour maps that are converted from the DEM generated by the aggregation method, resample method, W8D algorithm and VIP method are placed individually on the same layer with the contour map that is converted from the actual 1:50000 DEM. We can see from (a) to (d) that compared with other methods, the contours that represent the W8D algorithm are closer to the actual contour in the mountaintop area and the valley area at the top of the picture. There is little displacement, which is the same trend as the actual contour. The maximum and the minimum values are preserved better than with the other methods because the W8D algorithm considers the watershed as the basic unit and preserves all of the valley lines and ridge lines.

(2) Elevation statistical parameters

**Fig 15:**
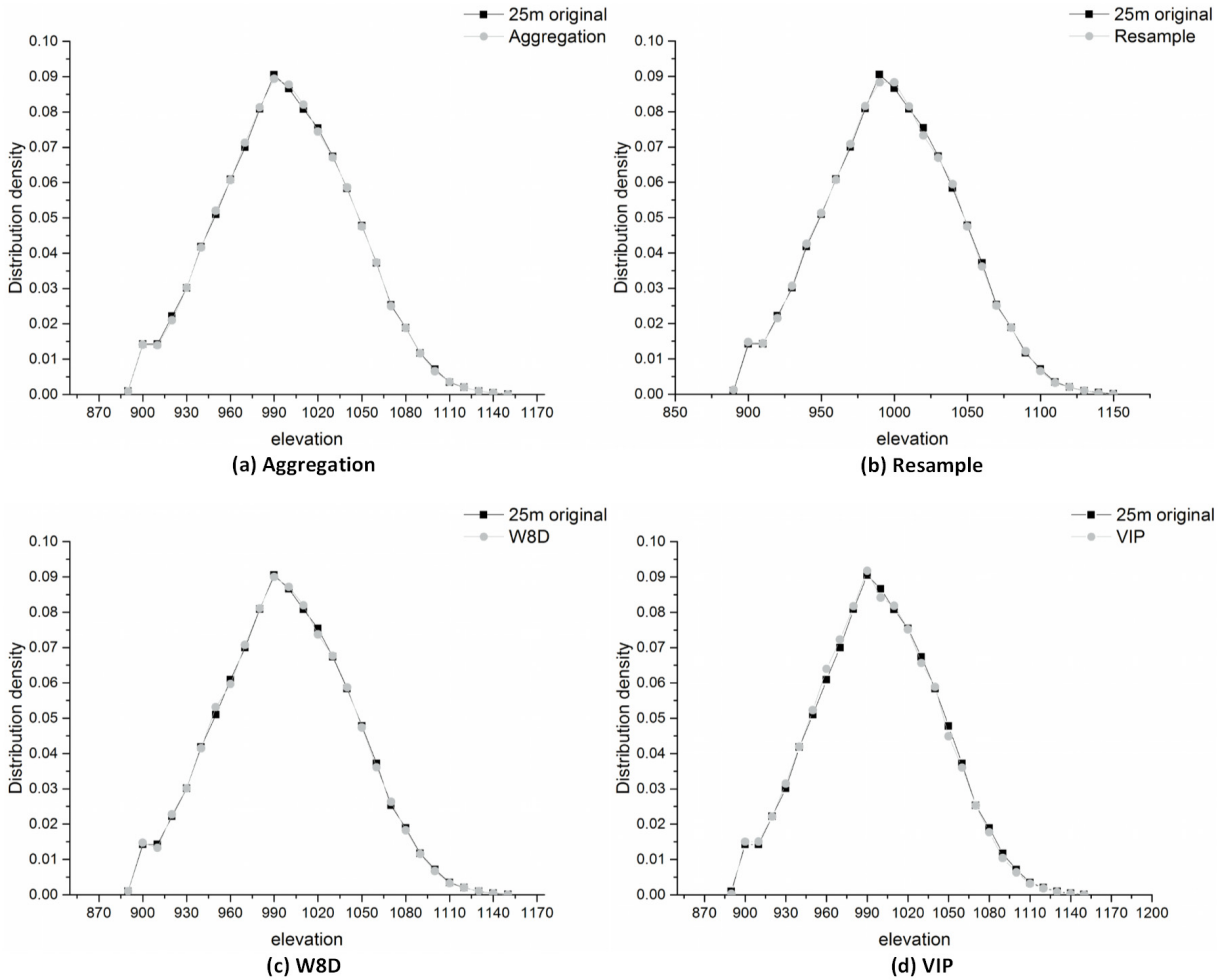
The comparison diagram of the distribution frequency of the elevation under 25 m resolution DEM.

A more direct method of assessment is to compare the elevation value of DEM before and after the generalization. Using this method, we can gain an overall picture of the effects that different generalization methods have on the DEM elevation value. Fig 15 shows that the distribution density of the elevation every 10 metres is different between the actual 1:50000 DEM and the DEM generalized from 1:10000 DEM. In this area, the cells whose elevation value is approximately 1000 m account for the largest proportion. Meanwhile, the distribution density of DEM that is generalized through the W8D algorithm is the closest to the actual DEM. Thus, the W8D algorithm performed well from an elevation point of view. The outcomes of the W8D algorithm are obtained through watershed internal generalization using 8-direction radial lines and the Douglas-Peucker algorithm. Thus, the data that the algorithm extracted are a subset of the feature elevation points in the original DEM. Hence, if we want the elevation distribution density to be closer to the 1:50000 DEM, we need to add more directions of radial lines, e.g., 16 or more, to increase the number of feature points to improve the accuracy.

(3) Slope and aspect analysis

**Fig 16:**
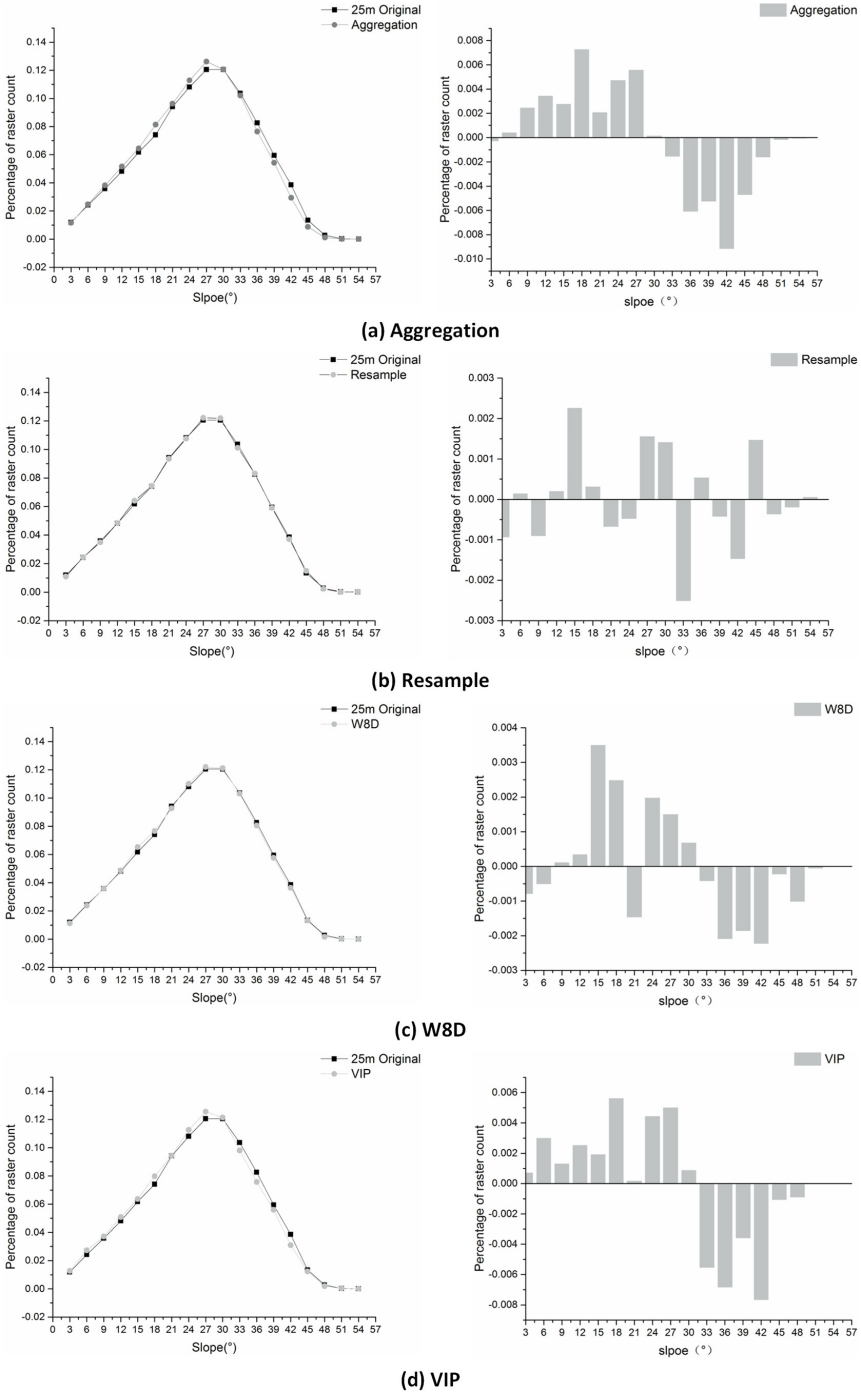
The comparison diagram of the slope frequency under 25 m resolution DEM.

Among the terrain feature factors, the slope gradient reflects the incline of the slope, the aspect reflects the orientation of the slope, and these two factors are the most important identifiers of topography. Liu noted that after the scale is converted, the statistical features, the spatial autocorrelation and the terrain structure features should be preserved as best as possible(Liu. 2007). Fig 16 is the comparison diagram between the slope distribution of the generalized DEMs that are computed by a 3° interval grading and the slope distribution of the actual DEM. The horizontal coordinate represents the slope value of every 3°; the vertical coordinate represents the amount of the grid and its proportion. Fig 15 shows the proportion differences of the grids in each grade between the generalized DEM and the actual 1:50000 DEM. The slope distribution features of the DEM that are generated by resample method and W8D algorithm are similar to the actual features. The method also has more correlativity. The differences in every grade in these two methods are below 0.5%. However, the difference in the aggregation method and VIP method is relatively larger, especially for the aggregation method, for which the biggest difference almost reached 1%. The reason for the difference is that in the study area, using the aggregation method and VIP method results in neglecting the feature points on the valley lines and ridge lines. However, the W8D method can preserve the feature points on the valley lines and ridge lines. The influences that different generalization methods have on the different landforms in terms of slope needs further study.

**Fig 17:**
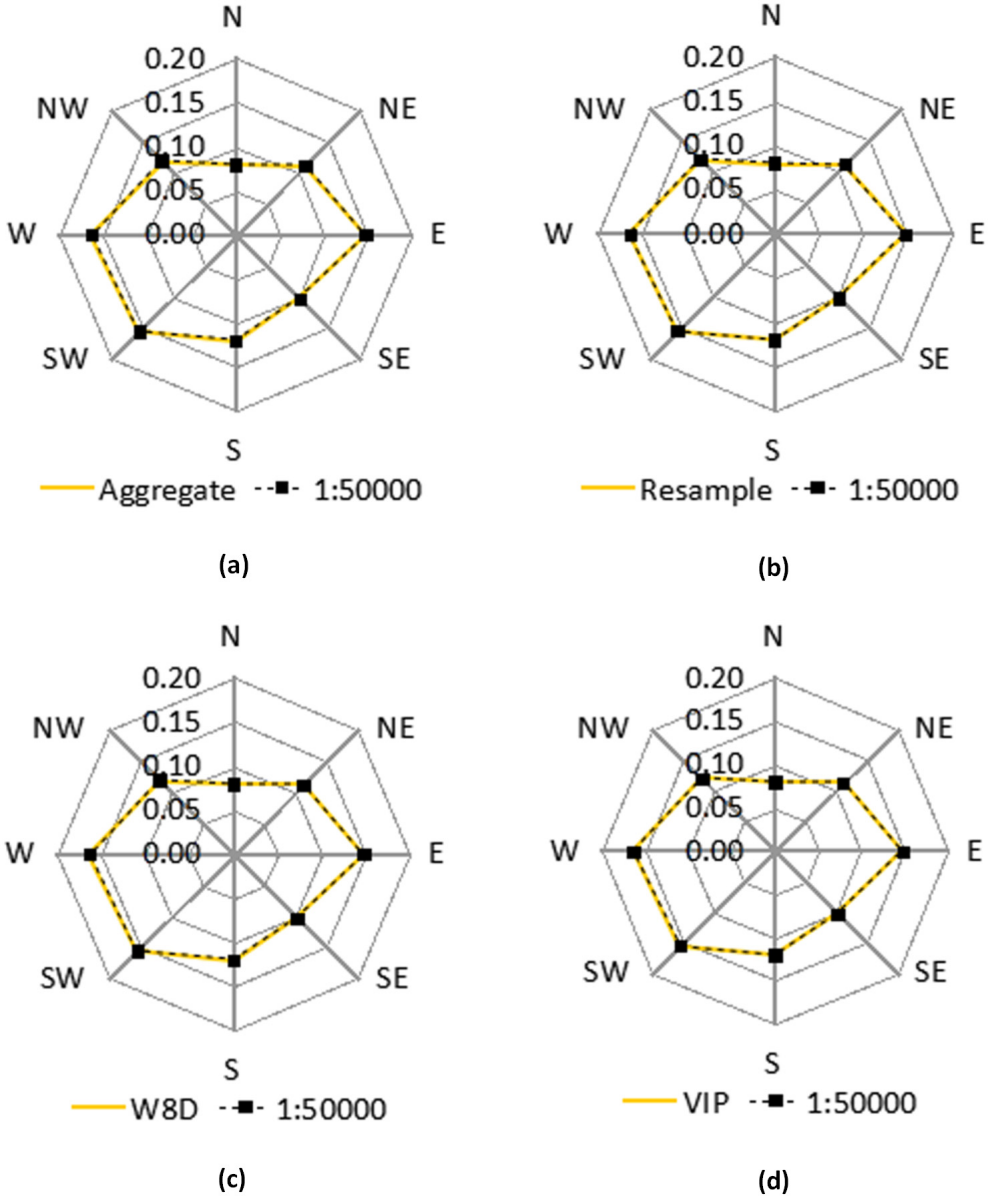
The radar graph of the aspect frequency.

Fig 17 shows the radar graph of the aspect frequency obtained by grouping the DEMs that are generated through different methods into 8 directions. The frequencies of the four generalization methods are almost the same as the actual 1:50000 DEM. The figure shows that the influence the different methods have on the aspect of the grids is relatively small.

In the areas that have fewer increases and decreases, a slight change in the elevation value of a single grid can lead to a change of aspect, which could lead to a big change in the aspect value of this point. In the gully areas, because of the rough terrain surface, a small difference in the elevation of a single grid does not have a significant effect on the aspect. Meanwhile, the wind rose diagram of aspect frequency is based on the eight directions. Because aspects are categorized into 8 groups, the aspect information of a certain range that might have a big change in a slight direction might be neglected; thus, the influence of this method might be neglected.

## 6. Conclusions and Discussion

This paper proposed a DEM generalization method based on watersheds and tree structures. The paper focused on the discussion of the threshold in the W8D algorithm using 1:50000 DEM as the target scale. Finally, the paper analyzed the effectiveness and usability of the W8D algorithm by comparing it with commonly used terrain generalization methods.

Firstly, compared with other classic methods, the processing cost of the W8D algorithm is expensive, and the computing speed is relatively low because a large number of points are located on the watershed border and a simplification process is conducted centred on each point. However, this problem can be solved by segmented images in other methods. However, this method is unable to solve the problem in the W8D algorithm. The algorithm will need further improvement to its computational efficiency for application to very large geo-spatial databases. Secondly, In this study we employed the simple D8 algorithm, which may not be the ideal method to retrieve streamlines from the original DEM [13]. The construction of a tree structure based on DEM is influenced by the accuracy of the DEM, the accuracy of terrain descriptions and the resolution of the DEM.

In the selection of best threshold, the paper used the 1:10000 DEM as the original data and the 1:50000 DEM as the target scale. By comparing the DEMs that are generated under different thresholds with the 1:50000 DEM on the mean value of elevation differences cell-by-cell, the paper determined that the accuracy is characterized by a U curve. The value showed a decreasing trend because the threshold is more suitable for a certain scale between 1:10000 and 1:50000. As for the 1:50000 scale, the best threshold is 0.06. If the target scale changes, it is most likely that the best threshold will also change. Through this method, a self-adaption terrain generalization method is achieved.

Through the preliminary experiment and discussion, the following conclusions can be drawn:

1. The implementation of the W8D algorithm is operable. It achieves the generalization purposes of multi-scales and is self-included. The algorithm can also better preserve the terrain features of ridges and valleys.
2. By studying the selection of the best threshold, the generalization achieved the purpose of selection according to need.
3. Through elevation comparison, overlapped contour contrast and slope and aspect analysis, the paper found that W8D performed very well.

## Supporting Information

S1 File Source code.rar. This is the our terrain generalization algorithm program and source code with C#.
(RAR)

S2 File Result_raster_threshold.rar. These file including our experimental results and DEM image under different threshold.
(RAR)

S3 File Analysis_data.rar. (RAR)
